# A Holistic Approach to a Dizzy Patient: A Practical Update

**DOI:** 10.7759/cureus.27681

**Published:** 2022-08-04

**Authors:** Ioannis Koukoulithras, Gianna Drousia, Spyridon Kolokotsios, Minas Plexousakis, Alexandra Stamouli, Charis Roussos, Eleana Xanthi

**Affiliations:** 1 Department of Neurosurgery, Faculty of Medicine, University of Ioannina, Ioannina, GRC; 2 Department of Physical Therapy, University Hospital, University of West Attica, Athens, GRC

**Keywords:** peripheral vertigo, chronic subjective dizziness, vestibular rehabilitation, cervical vertigo, meniere’s disease, central vertigo, vestibular neuritis, vestibular migraine, persistent dizziness, benign paroxysmal positional vertigo

## Abstract

Dizziness is one of the most common symptoms encountered by physicians daily. It is divided into four categories: vertigo, disequilibrium, presyncope, and psychogenic dizziness. It is essential to distinguish these four symptoms because the causes, prognosis, and treatment differ. Vertigo constitutes a disease of the central or peripheral nervous system. Central origin vertigo may be a life-threatening situation and must be detected as soon as possible because it includes diseases such as stroke, hemorrhage, tumors, and multiple sclerosis. Peripheral origin vertigo includes benign diseases, which may be fully treatable such as vestibular migraine, benign paroxysmal positional vertigo, vestibular neuritis, Ménière’s disease, and cervical vertigo. The HINTS (head impulse, nystagmus, test of skew) examination is essential to distinguish central from peripheral causes. A detailed history including the duration of vertigo (episodic or continuous), its trigger, and a clinical examination step by step following the appropriate protocol could help to make a definite and accurate diagnosis and treatment. Due to a lack of expertise in dizziness and inappropriate treatment, many patients are admitted to dizziness clinics with long-standing dizziness. A holistic treatment combining medications, vestibular rehabilitation, physiotherapy, and psychotherapy should be initiated to improve the quality of life of these patients. So, this review aims to recommend a clinical protocol for approaching a dizzy patient with vertigo and to present in detail the epidemiology, pathophysiology, symptoms, diagnosis, and contemporary treatments of all causes of vertigo.

## Introduction and background

Dizziness has been described as one of the foremost common medical conditions, affecting 15-35% of the overall population at some point in their lives [1]. In the United States, an estimated 7.5 million individuals with dizziness are examined in ambulatory care settings each year, and it is one of the most prevalent primary complaints in emergency departments [2]. Dizziness is often classified into four categories [1,2]: vertigo, disequilibrium without vertigo, presyncope (near-faint), and psychophysiological dizziness, which is commonly related to anxiety and panic.

Vertigo is a sensation of spinning or moving, either the person or the visual surrounding. Vertigo constitutes a disease of the central or peripheral nervous system [2]. In most cases, dizziness and vertigo occur in adult patients and a smaller percentage in young patients. These numbers explain why almost 20% of patients older than 60 years have experienced severe dizziness that affects their daily activities [2].

The differential diagnosis of vertigo is extensive and includes diseases of the central nervous system (CNS) and peripheral nervous system (inner ear). Vestibular symptoms originating from pathology in the cerebellum or brain stem are classified into central disorders [2]. Central disorders include life-threatening causes such as stroke, multiple sclerosis, tumors, and hemorrhage. These are suspected if the patient presents with associated neurological symptoms such as weakness, dysarthria, sensory changes, ataxia, or confusion. Conversely, symptoms arising in the inner ear or the vestibular nerve are peripheral [3]. Peripheral pathology is associated with nausea, vomiting, and hearing loss symptoms. In peripheral disorders, vertigo can be triggered by a change in the position of the head, stress, or trauma. The most common peripheral vertigo disorders are vestibular migraine (VM), benign paroxysmal positional vertigo (BPPV), vestibular neuritis, and Ménière's disease (MD). Therefore, it might be difficult to distinguish between central and peripheral causes in patients who present with vertigo as their only symptom [3].

Consequently, there are different causes of vertigo, including those arising from disturbances of the ear, nose, and throat (ENT), CNS, and cardiovascular system [4]. Another type of vertigo is due to a cervical spine disorder known as cervical vertigo. However, it remains unclear if cervical vertigo is an independent entity. Proponents of cervical vertigo usually believe it is the most common vertigo syndrome and confirm its diagnosis with a series of signs, symptoms, and tests, either irrelevant or inappropriate. At the same time, there is much evidence that cervical dizziness is a distinct disorder [4].

Overall, there are many different types of dizziness that affect patients’ daily activities. Therefore, this review aims to recommend a clinical protocol for approaching a dizzy patient.

## Review

A clinical protocol for approaching a dizzy patient

Dizziness is an atypical symptom that may arise from many diseases of the ear, nervous system, cardiovascular system, and psychiatric conditions. Also, many drugs used daily specifically in older people can cause dizziness such as antiarrhythmics, antiepileptics, narcotics, muscle relaxants, and anti-parkinsonian agents [5]. This leads to an extensive differential diagnosis.

The first pivotal step in the evaluation of patients with dizziness is to distinguish the three major symptoms associated with dizziness: vertigo, near syncope, and dysequilibrium. The sensation of spinning is associated with vertigo, the sensation of falling with dysequilibrium, and the sensation of fainting with near syncope episodes [6]. This paper aims to evaluate patients with vertigo, so the clinical protocol is based on these patients.

**Figure 1:**
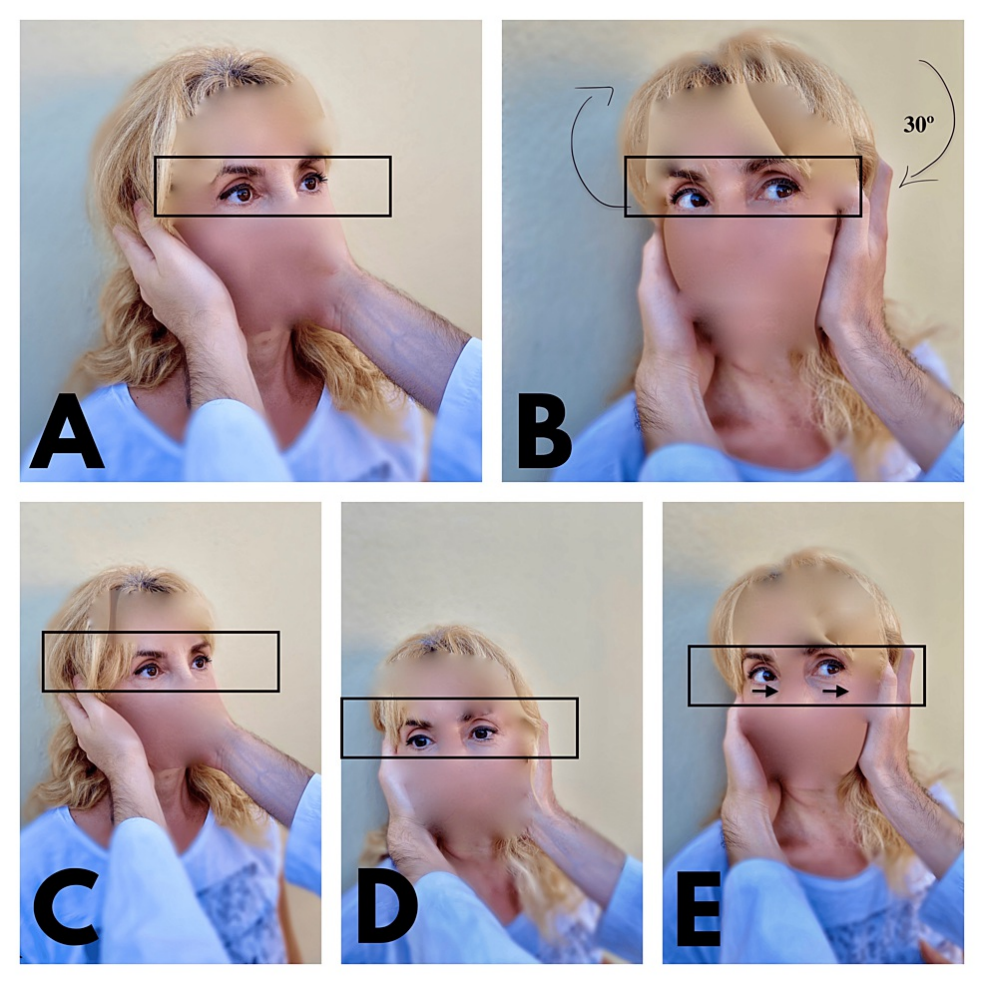
Head impulse test During this procedure, the physician turns rapidly the patient's head 30 degrees to the left or right and the patient should maintain the visual fixation (physician's nose). If central vertigo exists, the patient maintains the visual fixation (A, B). If visual fixation cannot be maintained during the head rotation and the eyes move with the head (saccadic eye movement), a peripheral vestibular lesion occurs (C, D, E).

**Figure 2:**
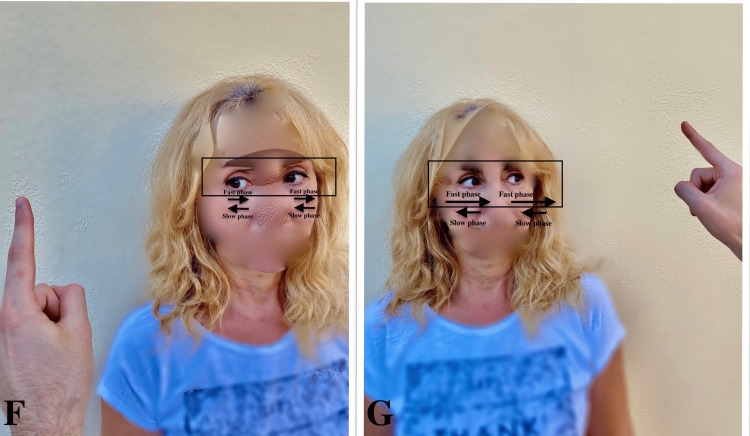
Nystagmus Due to acute (e.g. right) loss of vestibular function (e.g. vestibular neuritis), when the patient looks in the direction of the nystagmus fast phase (G), it increases and decreases in the opposite direction (Alexander's law) (F). It is well mentioned that the slow phase of nystagmus is always on the pathologic side and the fast phase is on the normal side. In central origin vertigo, a change of direction of nystagmus occurs.

**Figure 3:**
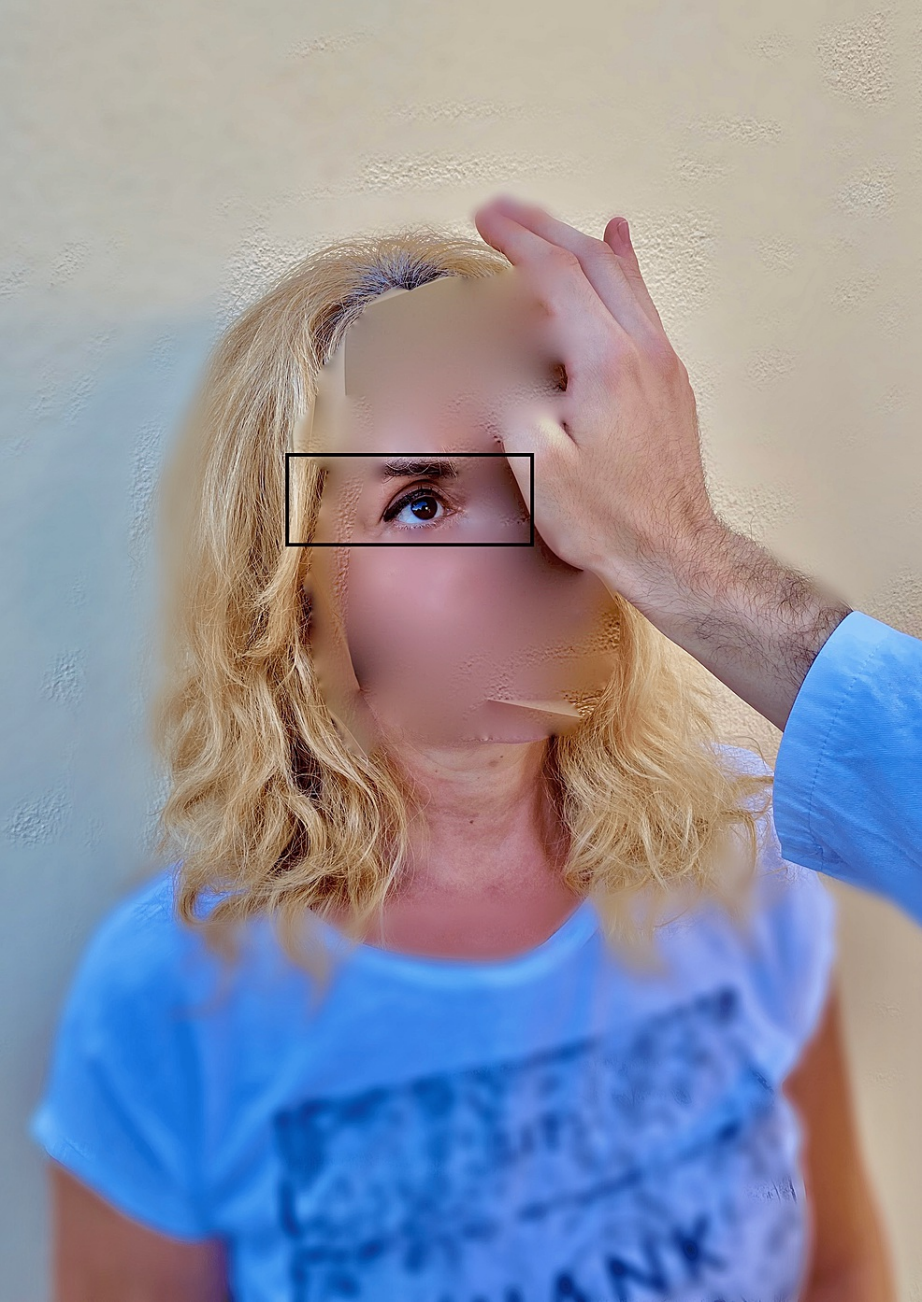
Test of skew During this procedure, the physician covers one eye of the patient and the patient is asked to fixate the vision to the examiner's nose. In central origin vertigo, the upper (hypertropic) eye moves downward when uncovered while the lower (hypotropic) eye moves upward for fixation. In peripheral origin vertigo, there is no skew deviation as presented in the photo.

The second pivotal step is to look for clues on history and neurological examination associated with CNS disease. Patients should be asked and examined for neurological deficits such as weakness, dysarthria, ataxia, incoordination, nerve abnormalities, and abnormal gait. Also, the characteristics of nystagmus associated with central origin are beneficial. These include nystagmus with the change of direction, nystagmus not inhibited by visual fixation, and nystagmus that lasts > one minute and fails to fatigue with repetition [6]. The HINTS (head impulse, nystagmus, test of skew) is a fast and near-bed examination that can distinguish central from the peripheral cause of vertigo with high sensitivity and specificity. Figures 1-3 present the HINTS examination [7].

The third pivotal step in patients without CNS symptoms evaluates the duration of vertigo (each episode of vertigo) as well as its trigger (triggered by head movements or worsen by head movements). In the patients, their vertigo is triggered by head motion (and absent while still), and the patients in whom the episodes last < one minute may have BBPV. A Dix-Hallpike test for the posterior semicircular canal or roll test for the horizontal canal can confirm the diagnosis [7].

The differential diagnoses of patients with recurrent episodes of vertigo that last for minutes to hours and worsen by motion but are spontaneous and present even without motion include stroke, VM, and MD.

**Figure 4:**
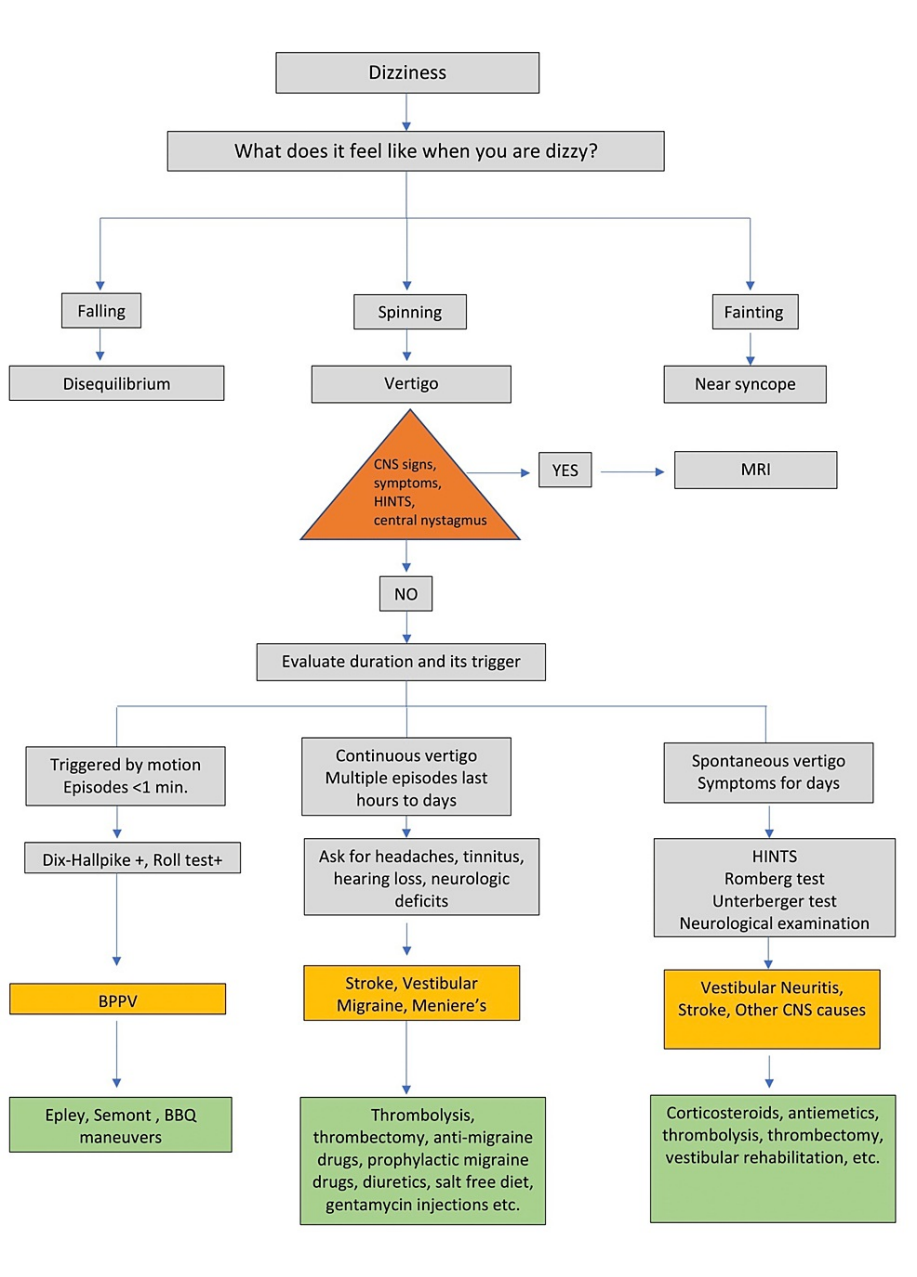
A protocol for approaching a dizzy patient HINTS: head impulse, nystagmus, test of skew; BPPV: benign paroxysmal positional vertigo.

Last, but not least, in patients with vertigo that is continuous and lasts for days, and is worse by motion, the differential diagnosis includes vestibular neuronitis, stroke, and other causes of CNS. Figure 4 presents briefly the steps that should be followed by a physician during the examination of a patient with dizziness.

The main causes of vertigo and their treatment are presented in detail below.

Benign paroxysmal positional vertigo

Many studies have revealed that BPPV is the most common cause of positional vertigo [8]. Patients complain about vertigo in certain head positions such as after lying down or sitting up from bed, turning from side to side, and in head extension. BPPV is the most successfully treatable cause of vertigo.

The exact mechanism is well known. Many causes such as age, head trauma, inner ear disease, vestibular neuronitis, osteoporosis, and surgery of the inner ear can cause dislodge of otoconia from the utricle to one of the three semicircular canals (canalolithiasis) [9]. Dix-Hallpike test can identify canalolithiasis of the posterior semicircular canal. The clinical presentation is torsional nystagmus after changes of head position. It is well mentioned that for approximately 50-70% of BPPV, the main cause is unknown [9]. Most common is the posterior canal BPPV (>80% of all positional vertigo). Table 1 presents the common diagnoses of positional vertigo with key features and treatments.

**Table 1:** Common diagnoses of positional vertigo with key features and treatments BPPV: benign paroxysmal positional vertigo.

Disorder	Symptoms	Nystagmus	Treatment
Posterior canal BPPV (>80%)	Vertigo (<30 sec) by turning head, lying down, sitting up. Symptoms for weeks, months, and years	Dix-Hallpike test. Mainly torsional nystagmus (fast phase in non-affected ear, slow phase in affected ear)	Epley maneuver (may need more than one session), Semont maneuver (alternative)
Horizontal canal BPPV (20%)	Vertigo (10 sec to 2 min) by turning in bed	Roll test. Horizontal (to ground - geotropic, to the sky -apogeotropic)	Barbecue maneuver (BBQ)
Anterior canal BPPV (seldom)	Vertigo (<1 min) when lying down, sitting up	Vertically downward	Epley maneuver
Central positional vertigo (vestibular nucleus, caudal cerebellum, etc.)	Variable duration of attacks, additional neurological presentations	Often pure upbeat, downbeat, change of direction	Underlying disorder

**Figure 5:**
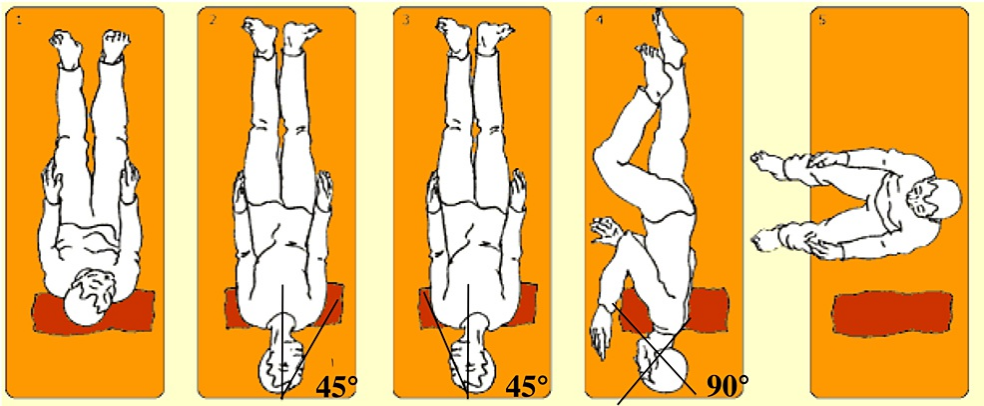
Epley maneuver (right ear) The procedure consists of a set of five head positionings that are hand-guided by a physician. Each positioning is performed rapidly and is maintained for 30 seconds. Sit the patient upright with head turned 45 degrees to the affected side (right) and then lie the patient down (1, 2). Rotate the head 90 degrees to the opposite side with the face upwards, maintaining a dependent position (3). Rotate the head and body another 90 degrees to the left (4). Raise the patient to a sitting position (5).

Epley maneuver has been established in a meta-analysis by Prim-Espada et al. (2010) as the most effective treatment for posterior canal BPPV (PC-BPPV) and is used daily in clinical practice [10]. Figure 5 shows the Epley maneuver.

Semont maneuver is another option although a multicenter randomized double-blind study has concluded that Epley was significantly more effective than the Semont maneuver for PC-BPPV [11]. Also, the vibration of the mastoid has been used during the maneuver, although it does not improve the outcomes significantly [5]. During the acute phase, antihistamines and anticholinergic drugs may be used to relieve nausea, vomiting, and vertigo such as dimenhydrinate, diphenhydramine, and metoclopramide.

Roll test can identify canalolithiasis of horizontal canal BPPV (HC-BPPV), which is the second most common cause of BPPV (20%), and the clinical presentation of patients is horizontal nystagmus, which can be geotropic if the nystagmus is toward the ground or apogeotropic if the nystagmus is toward the sky. Vertigo and nausea are more intensive than PC-BPPV. The affected ear is that with the more intensive nystagmus [5]. Barbecue (BBQ) rotation is a very effective maneuver for HC-BPPV [5].

Chronic dizziness

Nowadays, many patients admit to dizziness clinics with long-standing continuous dizziness. These patients have previously visited many doctors such as neurologists, ENT, and psychiatrists, and there is not a definite diagnosis. The expertise of dizziness is not well known and many doctors recommend symptomatic treatment without an exact diagnosis. Until 2017, there was not an original International Classification of Diseases (ICD) number for it. In 1986, there were descriptions of phobic postural vertigo followed by accounts of space-motion discomfort, visual vertigo, and chronic subjective dizziness from 1993 to 2004 [12]. Fortunately, in 2017, chronic positional vertigo was included in the ICD-11 by the World Health Organization [12].

Patients feel dizzy, have mild rotations, are a bit “drunk” or walk on a mattress or cotton wool, or are on a trip with the boat in waves. Also, these patients report that their symptoms worsen in “visually busy” surroundings such as walking through shelves in supermarkets, disco lights, and traffic. This is visual vertigo due to the sensitization of optokinetic stimulus (sensory conflict due to peripheral vestibular hypofunction) [5]. All patients with vestibular lesions are prone to be influenced by visual stimuli due to vestibular compensation. Visual desensitization techniques with vestibular rehabilitation methods should be initiated [5]. Also, many of these patients report that at some time they suffered from at least one vertigo attack. Detailed history taking reaches a diagnosis of BPPV, vestibular neuronitis, migraines, etc. Patients will make clear that they do not have rotational vertigo, but only a sensation of vague dizziness or unsteadiness [12].

Approximately the majority of patients with episodic vertigo (BPPV, vestibular neuronitis) will recover fully due to vestibular compensation [13]. However, patients with depression, anxiety, neurological deficits, and long-term administration of vestibular suppressants will be presented with chronic dizziness [12,13]. Table 2 presents the main conditions for chronic dizziness and its key features.

**Table 2:** Main conditions for chronic dizziness and its key features

Conditions	Clinical presentation
Bilateral vestibular failure	Oscillopsia during walking or driving, unsteadiness worsening in dark, head impulse test +
Neurological disorders (peripheral neuropathies, spinal cord syndromes, cerebellar, cerebral disorders)	Depends on the disease (tremor in Parkinson's, gait disorders, etc.)
Orthostatic hypotension	In elderly people (common) under antihypertensive drugs. Syncopal episodes, systolic blood pressure drops > 20 mmHg after sitting standing up
Chronic vestibular migraine	Low-grade dizziness, nausea, vomiting, migraine
Poorly compensated vestibular disorders such as vestibular neuronitis	Residual dizziness after the acute phase
Phobic, psychogenic vertigo	Psychological trigger of vertigo ("After this event, I have persistent dizziness")

The treatment of a chronic dizzy patient should be multidisciplinary. In the acute phase, many recommend vestibular suppressants (dimenhydrinate, diphenhydramine, diazepam, promethazine) and antiemetics (metoclopramide, scopolamine) [5]. It is well mentioned that these drugs should be prescribed only for three days because the long-term administration inhibits the vestibular compensation and chronic dizziness occurs. In chronic dizziness due to unilateral or bilateral vestibulopathy, vestibular rehabilitation is a very effective treatment with strong evidence [14]. So, counseling and rehabilitation, but no drugs, are the first-line treatment for chronic dizziness [15]. Also, vestibular suppressants may be used but not during vestibular rehabilitation. Cinnarizine is an antihistamine with mild antivertiginous action and strong antiemetic action. Last but not least, selective serotonin reuptake inhibitors (SSRIs) and serotonin-norepinephrine reuptake inhibitors (SNRIs) are very effective for persistent postural-perceptual dizziness (phobic vertigo) and patients can improve within one month [7].

Vestibular neuritis

Vestibular neuritis is usually caused by inflammation of the vestibular part of the eighth cranial nerve and it is ascribed to acute unilateral loss of vestibular function [16]. It is thought to be related to a prior or concurrent viral infection and the symptoms normally take weeks to months to resolve. It is worth mentioning that vestibular neuritis is not a life-threatening condition, identifying it through other conditions such as strokes is critical [17]. To differentiate vestibular neuritis from other central causes like central vertigo, BPPV, VM, psychogenic vertigo, MD, cervical vertigo, psychogenic vertigo, and chronic dizziness, an experienced physician is required to take detailed information about the patient’s history and conduct an appropriate clinical examination [17].

Epidemiology

The third most common cause of peripheral vestibular vertigo is vestibular neuritis, which accounts for 3.2 to 9% of the patients visiting a dizziness center and has an incidence of 3.5 per 100,000 population and an equal or near-equal sex distribution [18]. Vestibular neuritis affects people between the ages of 30 and 60, with the majority of cases occurring in people between the ages of 40 and 50 [19]. Also, it is more common in people over the age of 70, according to a recent study [19]. Superior vestibular neuritis is most common (55-100%), followed by inferior vestibular neuritis (3.7-15%) [20]. Acute vestibular neuritis or labyrinthitis is identified in 6% of patients who present to emergency departments in the United States with complaints of dizziness [16].

Symptoms

The most common symptoms are vertigo, nausea, vomiting, balance problems, and gait inconsistency [16]. Symptoms usually appear in the first few hours with a peak between 24 and 48 hours and last for several days before disappearing [21]. Symptoms are aggravated by head movements but not triggered by them [21]. Horizontal spontaneous nystagmus toward the non-affected ear with a rotational component associated with oscillopsia, a pathologic head-impulse test, a postural imbalance with a fall toward the affected ear, an incomplete ocular tilt reaction, an apparent horizontal saccadic pursuit, and gaze-evoked nystagmus toward the fast phase of the spontaneous nystagmus are all found during ocular motor evaluation [18]. A characteristic sign of these patients is that they usually prefer to lie in bed with their eyes closed in a side position with the healthy ear down [18].

Other signs and symptoms, such as headaches, are usually not present. It is important to ask the patient about any other symptoms that could indicate a central vertigo condition, such as visual or somatosensory abnormalities, weakness, dysarthria, incoordination, or inability to walk [21]. If any of these symptoms are present, the differential diagnosis must be expanded to include central causes of vertigo [21]. When unilateral hearing loss is present, the diagnosis is labyrinthitis [21]. When trying to differentiate between this hearing change and MD, keep in mind that MD can also cause vestibular and auditory problems [21]. Patients with MD, on the other hand, have more episodic symptoms that last 20 minutes to 12 hours [22].

Diagnosis

A vascular compromise of the peripheral vestibular labyrinth can potentially produce acute unilateral peripheral vestibulopathy [23]. Because imaging techniques cannot detect isolated labyrinthine infarction, diagnosis remains difficult [24]. Isolated labyrinthine infarction, on the other hand, is extremely unusual and frequently occurs in conjunction with cochlear injury and hearing loss [25]. Also, the infraction can occasionally spread to the brainstem or cerebellar area, supplied by the anterior inferior cerebellar artery (AICA) [10]. Infarctions of the vestibular nucleus or inferior cerebellum have also been linked to vestibular pseudoneuritis [24]. In patients with acute vertigo and nystagmus, the first clinical question to address is whether the symptoms are caused by vestibular neuritis or central vestibular pseudoneuritis [5]. A basic neuro-otological examination, which includes a normal horizontal head impulse test, direction-changing nystagmus, and skew deviation (HINTS), can consistently detect central vertigo with high sensitivity and specificity [26]. Even with high specificity, these bedside diagnostics are more sensitive to stroke than early MRIs [26].

Indeed, within the first 48 hours, 12-20% of stroke patients may have a false-negative diffusion-weighted MRI [24]. Because a mild degree of skew deviation is difficult to detect in the presence of spontaneous nystagmus, and gaze-evoked nystagmus may be absent in cerebellar stroke, the best technique for distinguishing isolated vascular vertigo from acute vestibular neuritis appears to be a bedside head impulse test [20]. Although a bedside head impulse test may be negative in patients with covert corrective saccades and inconclusive in individuals with nystagmus, video-based equipment recording of the head impulse test may be beneficial in patients with equivocal results [20]. A positive head impulse test, of course, does not rule out the potential of central lesions. Because recurrence of vestibular neuritis is uncommon, an alternate diagnosis should be considered whenever patients report multiple episodes [20].

Pathophysiology

The specific cause of vestibular neuritis is unknown. Vestibular neuritis is hypothesized to be caused by a viral infection of the vestibular nerve or ischemia of the anterior vestibular artery [27]. Recent research has shown an immune-mediated process as a cause of vestibular neuritis. Because associations with preceding or concurrent viral infection in the upper respiratory tract occur in 43-46% of cases of vestibular neuritis, viruses that cause infections of the upper respiratory tract, such as influenza virus, adenovirus, herpes simplex virus, cytomegalovirus, Epstein-Barr virus, and parainfluenza virus, are thought to be linked to vestibular neuritis [27].

The inflammatory response of peripheral blood mononuclear cells and the percentage of CD40-positive monocytes and macrophages are much higher in patients with vestibular neuritis than in healthy persons as a result of ischemia of the anterior vestibular artery [27]. Reduced microvascular perfusion of the vestibular organ is caused by an increase in thrombotic events caused by pro-inflammatory activation of peripheral blood mononuclear cells, CD40-positive monocytes, CD40-positive macrophages, and cytokines such as tumor necrosis factor-alpha, cellular adhesion molecule, and cyclooxygenase 2, which causes a loss of function of the vestibular organ secondary to reduced perfusion and/or infarction [27].

Treatment

Symptomatic treatment with antivertiginous drugs (e.g. dimenhydrinate and scopolamine) can be used to reduce vertigo, dizziness, and nausea/vomiting. Also, treatment with corticosteroids to improve peripheral vestibular function and physical therapy (vestibular exercises and balance training) to improve central vestibular compensation are all part of the treatment for vestibular neuritis [18].

Symptomatic Treatment

Vestibular sedatives, such as antihistamine dimenhydrinate 50 to 100 mg every six hours or the anticholinergic scopolamine, can be used during the first one to three days when nausea is severe. Sedation is the most common side effect. Scopolamine hydrobromide is used transdermally to avoid some of the negative effects associated with other methods of administration. Because these medications extend the time it takes to reach central compensation, they should not be used for more than three days [18].

Casual Treatment

Based on the hypothesis that vestibular neuritis is caused by the reactivation of latent HSV-1 infection, researchers undertook a prospective randomized double-blind experiment to see if steroids, antiviral medications, or a combination of the two may improve vestibular neuritis outcomes [28]. In 114 individuals, research with placebo, methylprednisolone, valacyclovir, and methylprednisolone plus valacyclovir found that monotherapy with steroids is enough to enhance peripheral vestibular function in patients with vestibular neuritis [28,29].

There was no evidence that methylprednisolone and valacyclovir worked together. Glucocorticoids (methylprednisolone) should be started three days after the onset of symptoms and continued for three weeks (at first 100 mg/day, then 20 mg every three days). The benefit of steroids may be attributable to their anti-inflammatory properties, which reduce swelling and mechanical compression of the vestibular nerve within the temporal bone [18].

As a result, steroids, but not antiviral medications, can be recommended as a treatment for acute vestibular neuritis since they improve functionally [18]. Prednisone medication, according to a recent prospective trial involving a small number of patients, may speed up recovery but may not improve the long-term prognosis of vestibular neuritis [18].

Physical Therapy

The central vestibular compensation of a peripheral deficit improves with a cautious program of physical training under the supervision of a physiotherapist. Static stabilization is prioritized first, followed by dynamic workouts for balance and gaze stabilization during eye-head-body movements. It is critical to gradually increase the difficulty of balance and equilibrium exercises above typical levels, both with and without visual stabilization. In a prospective, randomized, and controlled clinical trial, the efficacy of physiotherapy in improving central vestibulospinal compensation in individuals with vestibular neuritis was effective [18].

Central vestibular syndromes

Lesions of the vestibular pathways, which originate in the vestibular nuclei in the caudal region of the brainstem and travel to the cerebellum, thalamus, and vestibular cortex, or injury to the vestibulocerebellum are the most common causes of central vestibular disorders. Furthermore, central vestibular abnormalities can occur in the context of ocular motor disorders such as downbeat and upbeat nystagmus, episodic ataxia type two episodes, and VM [30].

Differentiating Between Central and Peripheral Vertigo

Severe imbalance, neurologic deficits, less prominent movement illusion, nausea, and central nystagmus (pure vertical/torsional, multidirectional, and no suppression with ocular fixation) are all common symptoms of central vertigo [31,32]. The essential information needed to distinguish between peripheral and central vertigo is usually found in the patient's medical history [31]. In peripheral vertigo, nausea and vomiting are usually more severe than in central vertigo. Vertigo is always connected with imbalance, and more severe imbalance is particularly linked to central causes. Patients with peripheral vestibular lesions exhibit the same imbalance as patients with central vestibular lesions, but they can walk. Many patients with central vestibular deficits, on the other hand, are unable to stand or walk.

In peripheral lesions involving the labyrinth or VIII nerve, auditory symptoms such as hearing loss, tinnitus, fullness, or discomfort in the ear are prevalent. In addition to hearing loss and tinnitus, internal auditory canal injuries have been linked to ipsilateral facial paralysis. Ipsilateral facial numbness, weakness, and limb ataxia can be caused by lesions in the cerebellopontine angle. Vertigo can also be detected as part of the aura of temporal epilepsy, and the patient is amnestic during the seizure. Acute vertigo caused by a peripheral lesion tends to improve in days to weeks due to a rapid compensation process, but central vertigo may not improve or improve slowly [31,33]. Optic fixation also inhibits peripheral spontaneous nystagmus, which is usually only noticeable for the first 12-24 hours [31,33]. Even with attention in the direction of the fast phase, peripheral spontaneous nystagmus can be entirely blocked in a few days. When the patient glances away from the fast phase, spontaneous nystagmus of central origin usually changes direction. It can last for weeks or months. A central vestibular lesion always causes vertical or pure rotatory nystagmus [31]. Diplopia, dysconjugate gaze, Horner syndrome, severe gait ataxia, dysarthria, dysphagia, facial weakness, numbness, long tract findings, and limb incoordination are all neurological indications that point to a central lesion. Diplopia, on the other hand, can be detected for a few days or so with an acute peripheral vestibular lesion due to deafferentation of otolith inputs [32]. A valuable finding to identify patients with unilateral vestibular hypofunction is head-shaking nystagmus, which is induced in response to a violent rotation of the head in the horizontal plane. The head-thrust technique, on the other hand, is employed to test the vestibulo-ocular reflex and it is only positive in the case of peripheral vestibular diseases. The patient fixes a visual target during the maneuver, and eye position is assessed immediately after a minor head thrust to the left and right. After the head thrust, a refixation saccade suggests a diminished vestibulo-ocular reflex on the side of the head thrust. The head thrust test will be positive in both directions if the fault is on both sides. When determining the cause of paroxysmal positional vertigo, postural testing is crucial. Caloric testing in peripheral vestibular diseases causes a reduced reaction in one ear, which is known as canal paresis. Both peripheral and central vestibular lesions exhibit directional preponderance. Defects in the conjugation of eye movements, saccadic pursuit, horizontal optokinetic abnormalities, central spontaneous or positional nystagmus, failure of fixation suppression, slowing of the nystagmus fast phases, perverted nystagmus, vertical optokinetic abnormalities, and retraction nystagmus are all symptoms of central vestibular disorders. General, neurologic, and neuro-otologic investigations, as well as screening tests for blood count, electrolyte and glucose levels, and thyroid function, are required in the case of unexplained vertigo. Last but not least, neuroradiologic imaging is indicated when a central lesion is suspected [31,34].

Vestibular migraine

Epidemiology

VM is more prevalent than other vestibular disorders. Neuhauser et al. found that the lifetime prevalence was 1% [35]. Meanwhile, the female-to-male predominance is about 5:1, with a mean age of onset of 37.7 years for women and 42.4 years for men [35,36]. Familial occurrence in some patients with an autosomal dominant pattern of inheritance has been reported. The majority of VM patients suffer from episodic migraine, and one in four of them have chronic migraine with medication overuse headache [35,37].

Symptoms

Many patients report internal vertigo (73%) and triggered vertigo, while external vertigo and positional vertigo are less frequent. One-third of patients experience unsteadiness. In almost 50% of patients, headaches accompany VM episodes; some report head fullness or head pressure without headache. Some patients report tinnitus, the fullness of the ear, palpitations, and mild and transient hearing loss, during their episodes. Several authors have described an accompanying Alice in Wonderland syndrome, a rare and fascinating sensory perception disorder, with the symptoms of visual distortions, extrapersonal misperceptions, or somesthetic distortions [37]. Like other vestibular disorders, along with vertigo, patients with VM may have phonophobia, osmophobia, photophobia, and visual or other auras. An aura is a temporary neurological phenomenon that appears before or during a headache. The duration of an aura can differ from a few seconds to several minutes or several hours and sometimes to a few days [37,38].

Pathophysiology

Although the pathogenesis of VM is still unclear, genetic, inflammatory, and neurochemical mechanisms have been proposed, mostly based on migraine pathophysiology, as migraine interacts with the vestibular system in many different ways [36]. Both the central and peripheral vestibular systems are associated with the pathogenesis of VM. First of all, many studies have revealed the overlap between vestibular and migraine pathways, as the caudal parabrachial nucleus receives both afferent peripheral trigeminal nociceptive and vestibular input. So, the cause of VM may be direct central activation of vestibular centers by the trigeminovascular system together with its effects on the inner ear. It seems that vestibular symptoms come from the vestibular nuclei, which are simultaneously suppressed by inhibitory feedback from the cerebellar nodulus and uvula [36,37]. Also, there is evidence of otolithic pathway abnormalities in individuals with VM. Moreover, studies have shown that Purkinje cells in the paraflocculus could be inhibited after a migraine episode, which is an important factor leading to VM. MRI studies have shown increased thalamic activation in VM patients during vestibular stimulation compared to healthy controls, as well as gray matter volume abnormalities of nociceptive and multisensory vestibular brain areas [37]. Another pathophysiological mechanism in VM may be a visuo-vestibular problem, as patients with VM have a longer duration of post-rotatory nystagmus compared to healthy people or only migraine patients. Last but not least, a genetic component in the pathogenesis of VM has been reported [36,37].

Diagnosis

The absence of any pathognomonic signs, blood, lab tests, and the presence of overlapped symptoms between the VM and the other causes of vertigo makes it more difficult to diagnose the VM. To diagnose VM, the physician must exclude all other causes of vertigo [38]. Neuhauser et al. suggested guidelines that are sub-divided into obvious and apparent VM criteria based on migraine diagnosis given by the International Headache Society. Recently, Lempert et al. published a document for the diagnostic criteria of VM used in daily clinical practice. VM must be differentiated particularly from MD and BPPV. Interestingly, some patients may experience the clinical features of both VM and MD during their attack. This has been described as "VM/MD overlapping syndrome." For differentiation, positional testing, which stimulates vertiginous attacks and elicits nystagmus, may be helpful, including the Dix-Hallpike maneuver or the supine roll test for the horizontal canal variant [36,37,39,40]. Other disorders that rarely mimic VM and should be taken into consideration during the differentiation process include central nervous system disorders such as vertebrobasilar transient ischemic attack and vascular compression of the eighth nerve [37]. Table 3 presents the diagnostic criteria of VM.

**Table 3:** Diagnostic criteria of vestibular migraine (VM) To diagnose probable VM, only one of the B and D criteria is required to be fulfilled.

Diagnostic criteria of VM
A. At least five episodes fulfilling criteria C and D.
	B. Current or previous history of migraine with or without aura according to International Classification of Headache Disorders (ICHD-3).
C. Vestibular symptoms of moderate or severe intensity, lasting between 5 minutes and 72 hours.	
	D. One or more migraine features with at least 50% of the vestibular episodes:
(i) Headache with at least two of the following characteristics: unilateral location, pulsating quality, moderate or severe pain intensity, and aggravation by routine physical activity
(ii) Photophobia and phonophobia
(iii) Visual aura
E. Not better accounted for by another ICHD-3 diagnosis or by another vestibular disorder.

Treatment

Due to the lack of well-conducted randomized clinical trials, VM treatment recommendations are mostly based on the guidelines for migraine therapy or observational studies, retrospective cohorts and open-label trials, anecdotal experience, and expert opinion [37]. Generally, migraine prophylactic treatments were effective in 77% of VM patients. Prophylactic medications are the mainstay for the management of VM. In a prospective study, Domínguez-Durán et al. reported that after five weeks of treatment, topiramate, amitriptyline, propranolol, flunarizine, and acetazolamide were all equally effective in reducing vestibular symptoms, headache, and the number of crises. Triptans like sumatriptan were reported to be effective when the vestibular symptoms were associated or not associated with a headache [35-37]. Other non-invasive treatments include lifestyle modification, trigger avoidance, and vestibular rehabilitation, although the results are inconsistent. Various treatment options are available for patients with VM, which include reduction of triggers and physical therapy. An abortive therapy is used to treat an acute attack of a headache or vertigo. The use of non-pharmaceutical methods such as vestibular rehabilitation in the management of VM is also helpful and in some patients, these approaches were found to be more effective than drugs [38]. Other possibilities include amitriptyline and calcium channel blockers. Clinicians could consider treating both or multiple illnesses, for example, nortriptyline for concomitant anxiety and topiramate or propranolol for associated headaches/hypertension. Physiotherapy and psychotherapy may play a role in treating some of the associated sequelae of VM, which include anxiety, visual dependence, and/or loss of confidence. Currently, no surgical management is recommended for VM [36].

Ménière's disease

MD is a disorder that affects the peripheral audiovestibular system. Even though the progression of the disease in most situations is slow, the impact on the social functioning of the individuals is important. The main symptoms of this MD are vertigo, tinnitus, aural fullness, and hearing loss. MD seems to have been caused by an amassing of endolymph in the cochlear duct of the inner ear [41].

Epidemiology

The prevalence of MD varies significantly depending on topographical and national aspects in addition to the environment in which the clinical assessment took place [42]. More specifically, the prevalence of MD seems to vary from approximately 3.5 per 10,000 to 513 per 10,000 patients and it appears to strike more often in white females who live through their fourth decade of life [42,43].

Pathophysiology

The exact etiology of MD is unclear yet. There are different theories about the development of MD, but environmental and genetic factors seem to play a role. Recent studies have shown that the development of MD is associated with anatomic changes in the inner ear. More specifically, studies have revealed endolymphatic accumulation in the cochlea and the vestibular organ. Endolymphatic hydrops (EH) may cause hearing loss up to more than 40 dB. Although EH is considered the histopathological hallmark of MD, it may reflect the final stage of a fluid imbalance in the cochlea and the saccule secondary to an inflammatory process of the cochlea with tissue damage that may occur at the organ of Corti, the stria vascularis, and the spiral ligament of the endolymphatic duct or sac. Recent research studies maintain that EH is a result of multiple etiologies. EH may be due to an autoinflammatory process, a viral infection, or a relapsing-remitting autoimmune disease [41]. In this way, fluid imbalance and tissue damage cause decay of the cochlea or genetic factors associated with developmental anomalies such as hypoplasia of the temporal bone. The radiological findings of the temporal bone propose that different endolymphatic sac pathologies (chronic inflammation leading to degeneration or hypoplasia) could be affiliated with different clinical subgroups of MD. Vertigo’s association is not clear yet. That is why EH is not entirely associated with MD. Instead, EH can be also found in cases of idiopathic sensorineural hearing loss [41,44].

Symptoms

The main symptoms of MD are vertigo, tinnitus, aural fullness, and hearing loss [41-46].

Diagnosis

The Bárány Society, the American Academy of Otolaryngology-Head and Neck Surgery (AAO-HNS), the Japan Society for Equilibrium Research, the European Academy of Otology and Neurotology (EAONO), and the Korean Balance Society have established an accurate clinical classification to diagnose MD [42-45]. Table 4 presents the diagnostic criteria for MD.

**Table 4:** Diagnostic criteria for definite and probable Ménière's disease

Diagnostic criteria for definite and probable Ménière's disease	
Definite Ménière's disease	Two or more spontaneous attacks of vertigo with each lasting 20 minutes to 12 hours.
	Audiometrically documented low- to medium-frequency sensorineural hearing loss in the affected ear, at least once, before, during, or after one of the vertigo episodes.
	Fluctuating aural symptoms (tinnitus, fullness, or hearing loss) are located in the affected ear.
	Other vestibular diagnoses were excluded by different clinical tests.
Probable Ménière's disease	At least two episodes of vertigo or dizziness, each lasting 20 minutes to 24 hours.
	Fluctuating aural symptoms (tinnitus, fullness, or hearing loss) in the affected ear.
	The condition is well explained by another vestibular diagnosis too.

These current diagnostic criteria are very useful to differentiate patients who suffer from a definite MD from those that suffer from a probable MD. The most significant finding of MD is low to medium frequency sensorineural hearing loss as mentioned above. Therefore, relevant history taking should be followed by an audiological evaluation. The hearing loss can progress to all frequencies with tinnitus being a common symptom and it is ipsilateral. These assessments are mandatory for the diagnosis of MD. Besides that, it is important to mention recurring and fluctuant characteristics of the hearing loss pattern. In addition, a fundamental diagnostic step both in the first stage and during the follow-up is the side-eye movement evaluation. New laboratory techniques have now enriched the methods to evaluate MD. Videonystagmography (VNG) allowed real-time meme observation of nystagmus with its third dimension [41,42].

The function of the utricle and saccule as well as the superior and inferior vestibular nerves are evaluated by vestibular-evoked myogenic potentials (VEMPs). VEMPs are the reflexes that are rising as a response captured through the sternocleidomastoid and orbital muscles owing to high intense acoustic stimuli. These can either be applied as bone conduction or air conduction to stimulate the otolith organs. Today, VEMPs are rather used for monitoring the otolith function and the effect of intratympanic gentamicin applications [41].

Patients with one-sided hearing loss should undergo MRI to exclude retrocochlear pathology. There is no need to perform imaging in the acute setting but it may be done within a few weeks after the beginning of symptoms as high resolution. There is a possibility that MRI imaging shows EH directly in the organs that have been affected. More research is ongoing to indicate if this is clinically useful. Vestibular (caloric) function testing may show a significantly under-functioning affected organ in 42-74% and a full loss of function in 6-11% [41,42].

Treatment

Preventive first-line management: MD patients are strongly recommended to receive a personalized approach. The modification of the lifestyle includes sleeping well and research on obstructive sleep apnea syndrome decreasing stress, avoiding alcohol, tobacco, and caffeine, and adopting a diet low in salt. A low sodium diet and high water intake may prevent the release of vasopressin and help to maintain inner ear homeostasis. The AAO-HNS scale limits caffeine in MD arguing that caffeine can cause changes in endolymph volume with its sympathomimetic action. There is an assumption that low amounts of caffeine will not trigger Ménière's symptoms. Furthermore, there have been remarkable studies about the efficacy of betahistine in reducing the vertigo episodes of MD. Some studies also suggest its dose-dependent effect in suppressing the frequency of vertigo attacks. The use of betahistine 48 mg twice daily for three to six months, according to clinical experience, is recommended to prevent Ménière's seizures. A meta-analysis by Nauta suggested the therapeutic benefit of betahistine in MD. Diuretics also represent the most commonly used first-line medical treatment. Chlorthalidone, acetazolamide, and hydrochlorothiazide are used in decreasing order. Their use is recommended to decrease vertigo spell frequency. Another non-invasive option is the Meniett® system (Medtronic, Dublin, Ireland), producing micro-press pulse sequences prone to act on the EH. Even though a recent review by Cochrane concludes that there is no evidence that this treatment is effective, the very low percentage of side effects reported in the five studies in the Cochrane Review recommend the use of this device as a first-line treatment [45,46].

Preventive second-line management: After using one or all of these treatment options, 80% of patients are in a relapse of MD symptoms, especially vertigo. To conservatively manage it in the remaining patients, intratympanic steroid injection (ITS) is recommended as a second-line treatment. In the last two decades, intratympanic treatment has been very popular. Dexamethasone is used more than methylprednisolone [45,46].

Third-line management: Endolymphatic sac surgery (ELSS) represents the third-line treatment of MD, even if it has long been criticized and considered a placebo surgery. Most critics referred to two placebo-controlled Danish studies analyzed in the Cochrane review in 2010 and 2013. Both studies concluded that ELSS had no documented effect on MD's physical course and vertigo. But a more recent meta-analysis concluded that there was a low level of evidence in favor of an outcome. Even if solid proofs are lacking in the literature, the authors agree that it should be the first option after failure of the medical conservative treatment, if the hearing function is useful and MD is in young subjects. All the authors favor the ELSS but the evolution of the practice toward an increased number of ITS, especially in the USA and in France, and a decreased number of ELSS surgeries [45,46].

Fourth-line management: Intratympanic injection of gentamicin (ITG) is probably the most effective non-surgical treatment to eradicate vertigo in MD and is an aminoglycoside antibiotic having more vestibulotoxic than cochleotoxic effect. Its effect is mainly causing atrophy in type 1 vestibular cells as well as the neuroepithelium. Although the intratympanic application of gentamicin carries the risk of hearing loss, many clinical trials have been designed to determine the lowest risk of its application with maximal vertigo control in MD [45,46].

Fifth-line management: Methods that have gained high evidence for MD’s treatment are labyrinthectomy and vestibular neurectomy. Among these two, vestibular neurectomy is a selective technique issued to superior and inferior vestibular nerves and keeps the cochlear nerve safe. The efficiency of both techniques is good. Ventricular neurectomy (VN) is believed to be the most effective technique for fall seizures (Tumarkin's disorder) and MD’s impotence. Labyrinth resection is the oldest surgical method for the treatment of MD and is currently limited to elderly patients. The technique may be associated with cochlear implantation at the same stage in the event of profound bilateral hearing loss. Cochlear implantation is recommended for surgical labyrinthectomy to rehabilitate hearing at the same time [45,46].

Cervical vertigo

Vertigo originating from the cervical spine is known as cervical vertigo. However, it is still debatable whether cervical vertigo is a separate entity. The pathogenesis of cervical vertigo is not clear yet but four hypotheses explain vertigo of a cervical origin including proprioceptive cervical vertigo, rotational vertebral artery vertigo, Barré-Lieou syndrome, and migraine-associated cervicogenic vertigo [4].

Proprioceptive Cervical Vertigo

The proprioceptive cervical vertigo hypothesis supports that damaged joint receptors in the upper cervical region may send abnormal afferent input to the vestibular nucleus, altering the vestibular nuclei of the brainstem and causing cervical vertigo. The most common clinical feature is upper cervical spine pain and dizziness. It frequently occurs as a result of whiplash injuries [4,5].

Rotational Vertebral Artery Vertigo

The rotational vertebral artery vertigo theory supports that during cephalic rotation, occlusion or insufficiency of the vertebral artery can cause decreased blood flow through the posterior inferior cerebellar artery, resulting in vertebrobasilar insufficiency and vertigo. Using color duplex sonography, a decrease in vertebral artery velocity and vertigo was discovered during head rotation. MRI or computed tomography angiography (CTA) can be used to detect compressive pathology in the vertebral arteries [4].

Migraine-Associated Cervicogenic Vertigo

The migraine-associated cervicogenic vertigo theory supports that reciprocal connections between the vestibular nuclei and the trigeminal nucleus caudalis may provide a mechanism by which vestibular signals influence trigeminal paths, which is closely linked to the processing of vestibular and trigeminal information during migraine attacks. Migraine was caused by a similar activation of the cervical trigeminal path, which then led to cervical vertigo. The main symptoms are cervical pain, stiffness, migraine, and vertigo [4].

Diagnosis

The diagnosis of central vertigo is very difficult, and it is only diagnosed after all other possible causes of dizziness or vertigo have been ruled out [47]. The symptom of neck pain is crucial in the diagnosis of cervical vertigo. Cervical vertigo is frequently misinterpreted as BPPV. As a result, a Dix-Hallpike test is required for vertigo sufferers [4].

Cervical vertigo is characterized by pain at the back of the neck and the occipital region, as well as stiffness in the neck. By bending the neck, symptoms can be triggered, and nystagmus can be caused, but not by simply putting the head in space [48]. Also, cervical vertigo is usually episodic, lasting minutes to hours at a time [4,47]. Moreover, the symptoms are frequently exacerbated by neck motions and they are alleviated by neck pain relief procedures [47].

Cervical vertigo can develop days, weeks, months, or even years after a head and neck injury [49]. Neck pain frequently radiates in a banana-shaped pattern to the temporal-parietal area and may only be felt when the neck is deeply palpated. As a result, some individuals may be unaware that they have neck pain until they are evaluated. Reproducible vertigo with neck manipulation, pain with palpation of the suboccipital region, cervical transverse processes of C1 and C2, cervical spinous processes of C2 and C3, levator scapulae, upper trapezius muscle, splenius, rectus, and semispinalis muscles are all exam findings in cervical vertigo. During an otolaryngologist's normal examination, these findings are frequently overlooked [47].

The use of magnetic reasoning angiography (MRA) or CTA is particularly beneficial in identifying vertebral arterial compressive pathology. Digital subtraction angiography (DSA) is the most reliable and important technique for determining the specific location of mechanical vertebral arterial compression and making a surgical choice, especially when the head is rotated (rotation and extension) [49].

Some researchers believe that the neck torsion nystagmus test can be used to diagnose cervical vertigo. The patient's head must be stabilized as the body is rotated. When neck proprioceptors are triggered, the inner ear structures must theoretically stay at rest [49]. With the neck torsion test, it has been shown that up to 50% of patients without cervical spine disease experience nystagmus [50]. A positive reaction (nystagmus) may be a manifestation of the cervical ocular reflex rather than a sign of disease [50].

Treatment

The treatment of cervical vertigo is difficult due to the lack of the main cause [49]. It is recommended that if vertigo is caused by proprioceptive dysfunction in the upper cervical spine, the treatment should be the same as for cervical discomfort [51]. Several studies have shown that manual therapy is useful in the treatment of vertigo caused by cervical dysfunction [49]. Reid et al. conducted a randomized controlled trial to test the effectiveness of a specific type of spinal mobilization known as sustained natural apophyseal glides (SNAGs), which was deemed to be of good methodological quality. At six and 12 weeks after therapy, they observed that the treatment group had a substantial reduction in dizziness severity and frequency, as well as lower Dizziness Handicap Inventory (DHI) ratings and less neck pain compared to the control group. As a result, they concluded that SNAGs are a safe and effective manual therapy procedure for cervicogenic dizziness and pain [52].

Several researchers advocate for the use of vestibular rehabilitation in the treatment of dizziness caused by cervical problems [51]. A combination of reflexes mediated by vestibular, ocular, and cervical sensory input has been used to achieve cervical spine stability and posture. In cases of cervicogenic dizziness, it is hypothesized that a well-integrated vestibulocerebellar system would be better able to compensate for the changed cervical sensory input [6]. When manual therapy and vestibular rehabilitation are combined, positive results have been observed in published case studies [53]. Last but not least, physical therapy is a useful therapeutic option for cervical vertigo [54].

## Conclusions

There are numerous causes of dizziness and the differential diagnosis is extensive and includes diseases from the inner ear, peripheral and central nervous system, cardiovascular system, and psychiatric conditions. Among the causes of vertigo vestibular migraine, BPPV, vestibular neuronitis, cervical vertigo, and stroke must be considered. The physician should take a detailed history of the patient and follow every step of the diagnostic criteria to make an accurate diagnosis. Most causes of vertigo are treatable and appropriate treatment can significantly improve the quality of life of patients suffering from vertigo.
